# Multidisciplinary stakeholder engagement in a type 2 diabetes comparative effectiveness study utilizing real-world data

**DOI:** 10.1017/cts.2024.666

**Published:** 2024-11-29

**Authors:** Elizabeth H. Golembiewski, Mindy M. Mickelson, Juan P. Brito, Victor M. Montori, Rozalina G. McCoy

**Affiliations:** 1 Knowledge and Evaluation Research (KER) Unit, Mayo Clinic, Rochester, MN, USA; 2 Division of Quantitative Health Sciences, Mayo Clinic, Rochester, MN, USA; 3 Robert D. and Patricia E. Kern Center for the Science of Health Care Delivery, Mayo Clinic, Rochester, MN, USA; 4 Division of Endocrinology, Diabetes, Metabolism, and Nutrition, Mayo Clinic, Rochester, MN, USA; 5 Department of Medicine, Division of Endocrinology, Diabetes and Nutrition, University of Maryland School of Medicine, Baltimore, MD, USA; 6 University of Maryland Institute for Health Computing, North Bethesda, MD, USA; 7 Department of Health Policy and Management, University of Maryland School of Public Health, College Park, MD, USA

**Keywords:** Patient and stakeholder engagement, type 2 diabetes, comparative effectiveness research, real-world data, patient-centered outcomes

## Abstract

**Introduction:**

Patient and stakeholder involvement enhances the conduct and applicability of comparative effectiveness research (CER). However, examples of engagement practices for CER leveraging real-world data (i.e., data from routine clinical practice) are scarce. Notably, these studies differ from traditional clinical trials in their technical complexity and minimal prospective data collection, posing unique challenges for stakeholder involvement. This paper describes patient and stakeholder engagement in a CER study of type 2 diabetes (T2D) medications using real-world data from a large administrative claims database.

**Methods:**

A Patient and Stakeholder Advisory Group (PSAG) was formed to guide study design, conduct, and dissemination. The PSAG (*n* = 12) included individuals with T2D, clinicians, health systems leaders, professional society representatives, and a payer representative. Members were surveyed post-study initiation to assess their participation goals and experiences to date.

**Results:**

PSAG members influenced key design and methodological decisions, including cohort selection and adding an aim focused on patient preference elicitation. Survey results indicated high satisfaction with engagement processes and a desire for ongoing involvement. Most PSAG members cited their main goals as impacting the lives of people with T2D and ensuring the research’s relevance to clinicians.

**Conclusions:**

Best practices for engaging stakeholders in CER using real-world data are underdeveloped. Our experience suggests that an inclusive, stakeholder-engaged approach enriches the research process and ensures diverse perspectives are integrated into study design and conduct. Ongoing efforts will focus on assessing long-term engagement outcomes and PSAG member satisfaction.

## Introduction

Choosing the right medication for type 2 diabetes (T2D) presents complex challenges affecting a broad spectrum of stakeholders, including individual patients, clinicians, health systems, professional organizations, policymakers, and payers. People with T2D must consider existing comorbidities, individual clinical and demographic factors, variable side effect profiles, and preferences for different administration modalities [1]. In addition, people with T2D must navigate variations in cost, insurance coverage, and availability of treatments. In turn, clinicians treating T2D must balance antihyperglycemic medication recommendations with risks of diabetes-related comorbidities. Clinicians also contend with regulatory burdens for different medication classes (e.g., prior authorization requirements), their own experiences with different medication classes, and the preferences of their patients [2,3]. Professional organizations, like the American Diabetes Association, also play a critical role in this ecosystem by setting guidelines and advocating for standards that shape treatment practices [4]. Finally, health systems and payers have a stake in identifying optimal T2D treatment strategies across diverse settings and populations, as T2D is among the most prevalent and costly chronic conditions [5]. In addition, numerous quality measures related to T2D management and outcomes are used for public reporting and value-based payment models [6], which may all be impacted by medication choice. Thus, improving medication management in T2D – and, ultimately, relevant health outcomes – demands coordinated efforts among all stakeholders to address these challenges.

Given the complexity of this landscape, including people with T2D and other stakeholders as partners in clinical research is increasingly seen as essential for making studies more relevant and patient-centered [7,8]. Central to this movement is the Patient-Centered Outcomes Research Institute (PCORI), which mandates stakeholder engagement in its research funding [9]. However, while the benefits of such involvement have been recognized for qualitative studies and interventional trials [10,11], its role in studies utilizing real-world data – data collected from routine health care sources like electronic health records and insurance claims – is less understood. Real-world data are valuable for their enhanced external validity and practical feasibility, particularly for comparing the effectiveness of treatments that are already in clinical use or for comparisons that would otherwise be infeasible [12].

However, effectively engaging patients and other stakeholders in studies using real-world data presents unique challenges, especially due to the technical and methodological complexities involved [10]. The present work aims to bridge this gap by describing the rationale for, development, and implementation of a diverse Patient and Stakeholder Advisory Group (PSAG) in the context of an ongoing PCORI-funded study using a large administrative claims database to examine the comparative effectiveness of T2D medications in patients with moderate cardiovascular disease risk [13]. By detailing the influence of stakeholder involvement on the study’s design, conduct, and preliminary findings, we aim to contribute insights into the role of stakeholder engagement in comparative effectiveness research (CER) utilizing real-world data.

## Materials and methods

### Research context

T2D is a significant public health challenge [14], demanding patient-centered care that balances evidence-based medication choices with individual patient preferences and circumstances. Despite consensus on metformin as initial treatment for people with T2D who do not have indicators of high risk for cardiovascular disease (CVD) or chronic kidney disease (CKD), subsequent therapy decisions remain complicated due to emerging drug classes and unclear comparative effectiveness of common second-line options, like GLP-1 receptor agonists (GLP-1RA), sodium-glucose cotransporter 2 inhibitors (SGLT2i), dipeptidyl peptidase-4 inhibitors (DPP-4i), and sulfonylureas [3,15]. This complexity is compounded by the interplay of T2D with CVD, CKD, and heart failure (HF), comorbidities that are significant contributors to the morbidity, mortality, and financial burdens associated with T2D [16].

Recent randomized controlled trials (RCTs) have demonstrated the benefits of GLP-1RA and SGLT2i in improving cardiovascular and kidney outcomes in individuals with T2D with established or high risk for CVD, HF, and CKD [3,17,18]. However, critical gaps persist in directly comparing these drugs, understanding patient preferences, and evaluating within-class medication differences in contexts left out from RCTs. This is particularly important for people with T2D who are at lower risk of CVD, for whom current treatment recommendations are less established [3]. Addressing these knowledge gaps is critical for patients and clinicians to engage in shared decision-making, for professional societies to develop guidelines that reflect scientific evidence and patient priorities, and for health systems and payers to make formulary decisions.

To this end, the goal of the overarching study (ClinicalTrials.gov identifier: NCT05214573) is to compare the effectiveness and safety of second-line antihyperglycemic medications in adults with T2D at moderate risk for CVD through analyses of observational real-world data [19]. The real-world data used in this study was composed of an exceptionally large and diverse sample of unselected patients with private, Medicare Advantage, and Medicare fee-for-service health coverage included in the OptumLabs® Data Warehouse. By examining major adverse cardiovascular events, HF hospitalizations, and other patient-centered outcomes, and incorporating patient preferences into treatment effect estimations, the overall study endeavors to inform personalized T2D management strategies, contributing to optimized patient outcomes and healthcare decisions [19].

### *Patient and stakeholder engagemen*t

#### PSAG formation

A Patient and Stakeholder Group (PSAG) for this study was established to ensure that the design and conduct of the study were aligned with the experiences, needs, and priorities of individuals involved in the management of T2D. The group composition and functions were designed to be responsive to PCORI’s Patient and Stakeholder Engagement Rubric, which details when and how engagement can occur while planning and conducting the study and disseminating its findings [20].

**Figure 1.**
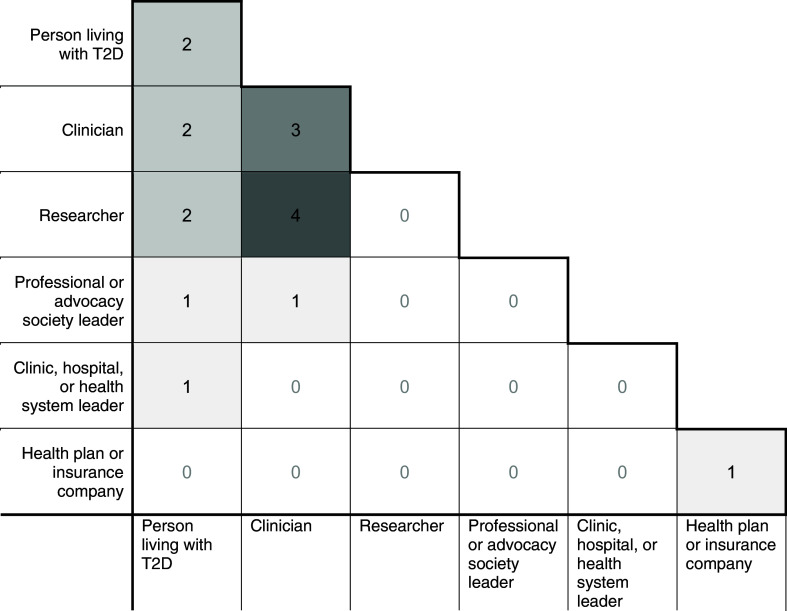
Role overlap among Patient and Stakeholder Advisory Group (PSAG) members (*n* = 12) for a type 2 diabetes comparative effectiveness study.

Potential PSAG members were identified and approached by the study principal investigator (PI) with input from co-investigators to ensure diverse experiences and perspectives that would complement those of the study team. The PSAG ultimately consisted of 12 members, all of whom accepted participation when approached by the PI. Demographically, the PSAG is 91.7% White and 16.7% Hispanic or Latino, with members’ ages distributed as follows: 16.7% are 35 – 44 years old, 25.0% are 45 – 54 years old, 25.0% are 55 – 64 years old, and 33.3% are 65 years or older. The group is predominantly male (75%), and the majority hold professional degrees (75%). These characteristics reflect a relatively homogeneous group in terms of race, gender, and educational background; however, members bring a wide range of professional and lived experiences that were critical to the study’s success. Specifically, the PSAG includes members with the following primary roles: three people with lived experiences of T2D; four clinicians who treat people with T2D; two health systems leaders; two leaders from relevant professional societies; and one payer representative. In addition, most members identified with more than one role relevant to the project (Figure 1).

PSAG activities (including the member survey, which is described in more detail below) were deemed exempt from continuing review by the Mayo Clinic Institutional Review Board.

#### PSAG member responsibilities

In agreeing to participate in the study’s PSAG, members were asked to make the following commitments:

- Attend semiannual meetings.
- Engage with the study team as able between meetings.
- Share their experiences, thoughts, and feedback openly.
- Assist in identifying barriers and opportunities to improve the research and its relevance to the communities they represent.
- Advise and assist in the dissemination of findings as they become available, including as coauthors on peer-reviewed manuscripts and conference abstracts.

PSAG members were also invited to volunteer to become more heavily involved in any specific study activities of interest, including contributing to study design and authorship of study publications.

In recognition of these commitments, PSAG members receive remuneration of $2,000 USD per year, with the exception of the three patient-investigator members, who received $3,000 USD in Year 1 due to additional expectations regarding protocol development and testing for the second aim of the study and $2,000 USD in Years 2 – 3 of the study, respectively.

#### Structure and activities

A comprehensive PSAG kickoff meeting took place virtually in September 2021. At this initial meeting, study team investigators were introduced and all teams were identified with stated team roles, professional identities, and study responsibilities. The PI and other members of the research team presented an overview of the study, focusing on its main goals, current challenges in managing T2D, and PCORI’s role as both collaborator and funder.

The PSAG has met and continues to meet with the study team every six months. Meetings are spent reviewing the preceding six months’ activities and plans for the next period, with a strong focus on eliciting and integrating feedback from PSAG members. This feedback, informed by their diverse backgrounds and experiences with T2D management, is crucial for improving the study and ensuring its relevance in practice. Facilitated by the study PI and project manager, these sessions are interactive and use first names to foster an atmosphere of respect and inclusivity. To ensure an accurate record, meetings are recorded and securely stored, although members can opt out of the recording and provide feedback at a later time. After each meeting, detailed minutes, presentation slides, and other relevant resources or materials are circulated for reference and for the sake of PSAG or study team members unable to attend.

The study team has also implemented several engagement measures to ensure that PSAG members, including those without formal research backgrounds, could feel empowered to contribute meaningfully. These measures include using accessible, lay language in presentations, illustrating concepts through real-world clinical applications and examples, and offering one-on-one follow-up discussions to clarify any uncertainties. Specifically, PSAG members were encouraged to contact the PI or project manager with any questions or concerns between meetings, and the project manager worked closely in particular with patient-investigators, who did not have formal research training, to ensure comprehension. Finally, the study team engaged with individual PSAG members or specific groups (e.g., clinicians only) as needed outside of regular meetings to obtain feedback on targeted items or concerns.

Overall attendance at semiannual meetings was high, with approximately 70% of PSAG members on average present across 6 total meetings. Attendance by role varied, with clinicians and researchers representing 83% of the time, followed by health systems representatives (75%), payers (67%), and patients (50%). Of note, overall patient attendance was relatively low because one of the three patient-investigators works in the maritime industry and has long stretches of time offshore without Internet access. The project manager worked closely with this individual to ensure access to and comprehension of study activities and PSAG meeting discussions.

#### Member survey

To understand PSAG members’ goals and perceptions of the engagement process, the study team developed and fielded an optional electronic survey from December 31, 2021, to February 13, 2022 (after the kickoff meeting and before the second PSAG meeting). Survey items were designed by the study team to align with the study’s Patient and Stakeholder Engagement Plan (submitted to PCORI as part of the grant proposal) and incorporated relevant items from existing public surveys and previous PCORI-funded studies where possible [21]. PSAG members were informed that responses would not be anonymous, allowing the team to address specific needs and suggestions directly, but that their participation was optional. The survey was fielded in Qualtrics, a secure online platform, with an option for respondents to complete it over the phone with the study’s project manager if preferred. Descriptive statistics of survey responses were computed using IBM SPSS Statistics (Version 29.0) and reported as counts and percentages for the overall sample and by stakeholder groups. No further quantitative analyses were conducted given the small sample size and selected nature of the respondents.

## Results

### PSAG influence on study activities

#### Overview

During PSAG meetings, study investigators actively sought feedback from all members, ensuring that each member’s unique perspective – whether as a person with T2D, a physician, a pharmacist, or a health plan decision-maker – was solicited. If specific perspectives did not emerge organically, the study team encouraged members to reflect based on their lived or professional experiences. For example, PSAG members were asked to reflect on how findings might be interpreted by different audiences, such as clinicians, payers, and patients, and asked for guidance to ensure the study design and conduct was relevant for these diverse groups.

At times, the study team observed potentially divergent perspectives between PSAG members, highlighting the value of such diversity. For instance, discussions arose about whether medication selection should prioritize the most effective option based on clinical guidelines (as emphasized by many clinicians in the group) or if it should be driven primarily by factors such as cost and treatment burden, which were priorities for patient-investigators.

#### Study design

All PSAG members reviewed the initial grant application and provided input on the specific aims and methods used to address the overarching question of what class of second-line glucose-lowering medications is best suited for people with T2D who are at moderate risk for CVD. Discussions at this stage centered on defining the patient cohort and, in particular, how to classify “moderate” CVD risk and whether to include patients both with and without metformin therapy at baseline. Patient-investigators, clinicians, and health plan representatives from the PSAG recommended developing and using a claims-based CVD risk score, as there were no alternative models that could be operationalized within the type of data available (i.e., claims data, rather than electronic health records). PSAG members also recommended the inclusion of both metformin-treated and nontreated groups in the study to ensure the generalizability and relevance of findings to their communities.

Additionally, patient members of the PSAG suggested adding a new, second aim to evaluate the comparative effectiveness of T2D medications across a broader range of outcomes than those listed in the PCORI funding announcement. The goal of this additional aim was to conduct a mixed methods participatory ranking activity among patients with T2D to generate evidence that could be maximally responsive to the diverse, individualized priorities of people living with T2D. Finally, payer and clinician members of the PSAG advocated for conducting within-class comparisons, in addition to across-class comparisons, in order to generate practical guidance on selecting medications within specific therapeutic categories, resulting in the third aim of the proposed study.

#### Study conduct

PSAG members provided critical input on several operational decisions of the study as it was conducted. These included: 1) quantifying the specific range of major adverse cardiovascular event rates that would define “moderate” CVD risk for the study; and 2) specifying which risk factors to include in the Annualized Claims-Based Major Adverse Cardiovascular Events Estimator model [22]. Furthermore, patient members of the PSAG were involved as co-investigators to develop and field test an inventory of T2D outcomes and medication attributes for a ranking exercise conducted with people living with T2D, with the goal of understanding how people with T2D prioritize treatment decisions [1]. More generally, PSAG members have helped the study team interpret findings and contextualize them in the broader landscape of T2D care.

#### Dissemination

To date, all peer-reviewed journal articles and conference presentations that have been produced from this study have PSAG members as coauthors, including as first authors. Specifically, PSAG members were included as coauthors on manuscripts where their expertise and contributions were directly relevant to the study or findings being disseminated. Authorship was not automatically extended to all PSAG members for every manuscript. Instead, authorship decisions were made based on individual input and engagement in the project’s various phases. Professional members of the research team or PSAG (i.e., researcher or clinician members) typically assumed first authorship roles, reflecting their central involvement in study design, data analysis, and manuscript preparation. However, patient-investigators have been included as coauthors where their insights or feedback significantly informed the research, particularly in areas related to patient perspectives or study relevance to community stakeholders.

### Findings from PSAG member survey

All 12 PSAG members completed the member survey that was fielded after the project kickoff meeting. Given the small sample size, survey results should be interpreted as descriptive rather than comparative, though differences between stakeholder groups are highlighted where relevant. Half of the members reported previous experiences participating in a study advisory group. A majority of members (83%) reported that their primary goals for participating were to impact the lives of people with T2D and to ensure that the research and its findings are relevant to clinicians, respectively (Table 1). PSAG members with T2D in particular prioritized making an impact on the lives of people with T2D (100%) and ensuring the research was relevant to people with T2D (80%) compared to other groups.

**Table 1.** Member goals for participation in a Patient and Stakeholder Advisory Group (PSAG) for a type 2 diabetes (T2D) comparative effectiveness study, overall and by role (*n* = 12)

Goal	Total	Persons living with T2D	Clinicians	Researchers	Health system, professional society, and payer roles
Making an impact on the lives of people living with diabetes	83.3%	100.0%	75.0%	83.3%	80.0%
Making sure that the research and its findings are relevant to *clinicians* who care for people living with diabetes	83.3%	80.0%	87.5%	66.7%	100.0%
Making sure that the research and its findings are relevant to *people* living with diabetes	66.7%	80.0%	50.0%	66.7%	60.0%
Making sure that the research and its findings are relevant to health systems	66.7%	60.0%	75.0%	66.7%	80.0%
Making sure that the research is done well	66.7%	80.0%	62.5%	83.3%	40.0%
Helping share information learned in this research study with others	66.7%	80.0%	62.5%	100.0%	60.0%
Being part of this research study as a co-investigator	50.0%	80.0%	50.0%	66.7%	20.0%
Connecting with others who have similar personal or professional interests	50.0%	40.0%	62.5%	83.3%	60.0%
Making sure that the research and its findings are relevant to insurance companies and payers	33.3%	0.0%	37.5%	50.0%	60.0%
Learning more about the research process	25.0%	40.0%	12.5%	33.3%	0.0%
Improving the experience of research participation for other people like you	16.7%	40.0%	12.5%	33.3%	0.0%
Seeking opportunities for professional growth and development	16.7%	20.0%	25.0%	33.3%	20.0%

*Note:* Respondents could select all that apply from a pre-specified list of statements. T2D = Type 2 diabetes.

**Figure 2.**
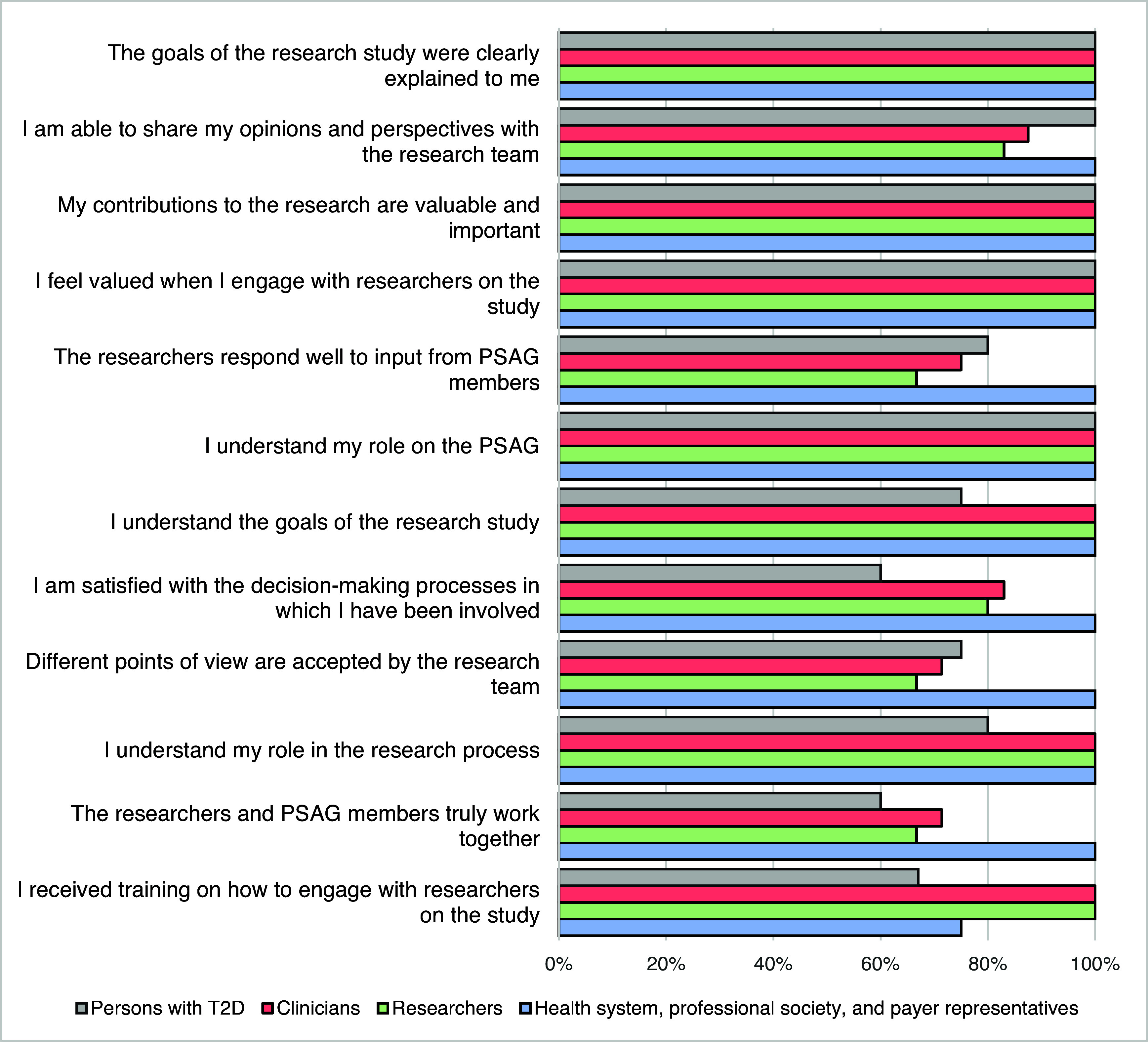
Level of agreement by Patient and Stakeholder Advisory Group (PSAG) members with statements regarding their experience in the PSAG, by role (*n* = 12) *Note:* The number of total respondents for each item varied from *n* = 11 to *n* = 12. Percentages reflect the proportion of respondents who strongly or somewhat agreed with a given item out of the total number of respondents for that item. T2D = Type 2 diabetes.

Survey results showed full agreement among PSAG members that the study’s goals were communicated clearly to them by the study team (Figure 2). In addition, most members reported feeling empowered to share their opinions (92%) and that they believed their contributions were valuable (83%). Satisfaction was also high with respect to member understanding of their roles on the PSAG (83%) and the decision-making processes they were involved into date (83%). However, lower proportions of members felt that the researchers and PSAG members were effectively working together (73%), especially among members with T2D (60%). In addition, there was some variability between groups in whether different points of view were accepted by the research team, with greater agreement among members from health systems, professional societies, and payer organizations (100%) and members with T2D (75%) compared to researchers (67%) and clinicians (71%). Finally, smaller proportions of persons with T2D (67%) and health systems, professional societies, and payer representatives (75%) agreed that they had received sufficient training on engaging with the research team compared to researcher or clinician members (100%).

**Figure 3.**
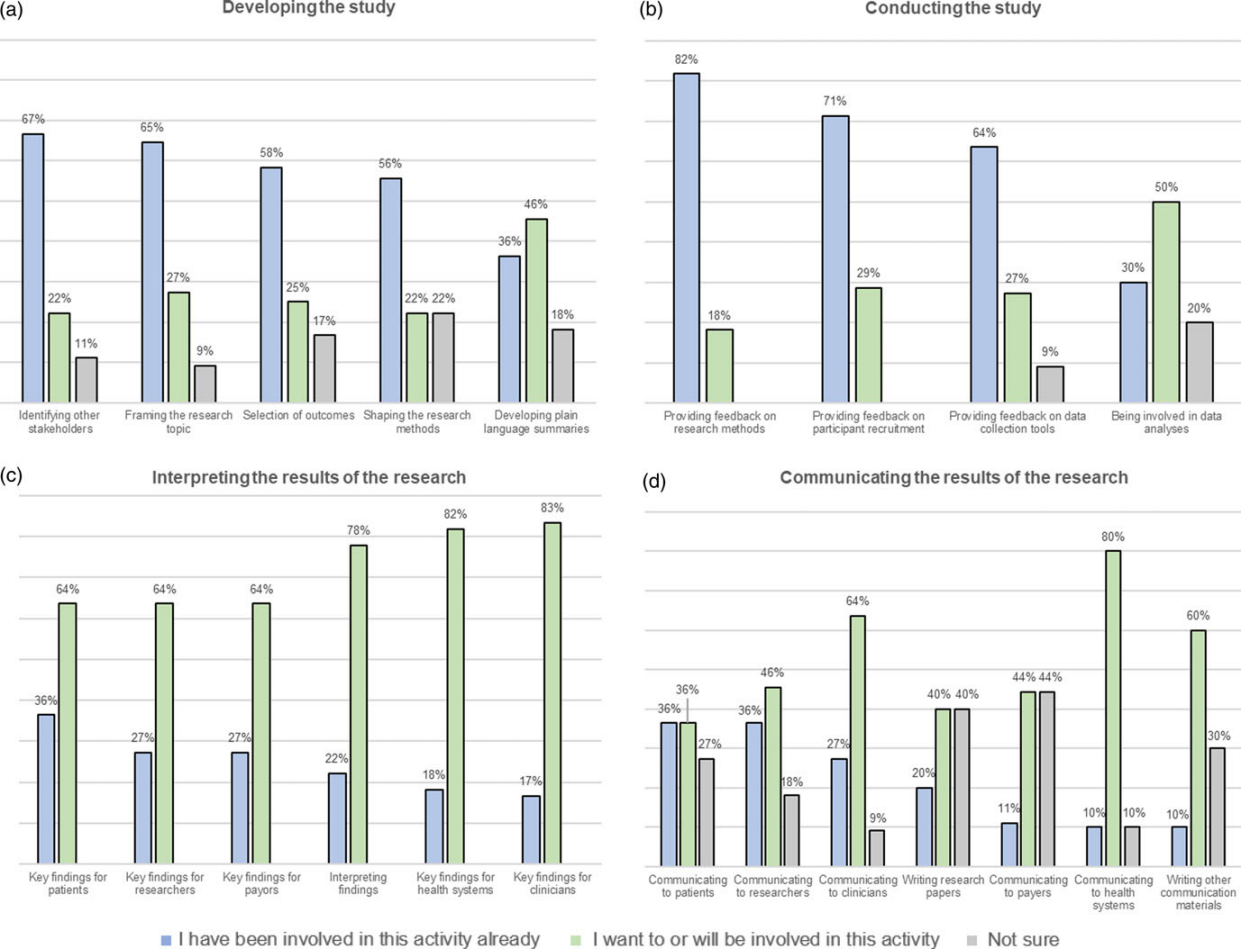
Patient and Stakeholder Advisory Group member self-reported involvement in current and future research activities by phase of study (*n* = 12).

At this early stage of the study, a substantial proportion of PSAG members felt they had actively been involved already in framing the research topic (65%), providing feedback on the research methods (82%), selecting outcomes to be studied (59%), and providing feedback on data collection tools (64%; Figure 3). There was also a strong interest among most members in participating in the interpretation of future research findings, especially those relevant to clinicians (83%) and health systems (82%).

Survey results were shared with the larger group at the March 2022 PSAG meeting. In response to feedback from the survey findings, the study team announced that a monthly newsletter would be issued to keep continuity in the discussion and allow PSAG members to remain up to date on study progress between the semiannual meetings. The monthly newsletters were also intended to identify any new or ongoing challenges, highlight accomplishments, and share additional engagement opportunities for PSAG members.

## Discussion

Decision-making in T2D medication management poses challenges to many stakeholder groups due to the evolving evidence base on medication efficacy and safety, heterogeneous treatment effects among patient subgroups, and differences in medication costs and availability. As a result, engaging stakeholders from multiple perspectives of this complex issue – including patients, clinicians, health systems leaders, policymakers, and payers – in all phases of the research process is foundational to ensure that the findings are relevant and that solutions proposed are actionable, pertinent, and meaningful. A limited number of previous studies on stakeholder engagement in T2D research have suggested the value of such engagement in enhancing the patient-centeredness and relevance of the research [23–26]. However, further insight was needed into how diverse stakeholders, impacted by disparate aspects of the complex ecosystem of T2D medication management, can collaborate to inform research that is itself complex and falls outside more familiar approaches like prospective controlled trials, like those that use real-world data.

Ironically, studies using real-world data may be more relevant for a wider range of stakeholders than clinical trials, because the data used in these studies reflects the conditions of routine healthcare; in other words, the settings in which patients are treated, health care professionals administer care, and health systems, organizations, and payers operate [27]. Stakeholders are also representative of the people indirectly contributing data through their roles in the health care system as patients, clinicians, or service providers. This highlights a critical area for development, aiming to harness the full potential of real-world data while aligning research with the needs and perspectives of its intended beneficiaries. Moreover, observational studies that utilize real-world data can be complex and methodologically intricate, posing a challenge for stakeholders who may not have extensive training in advanced statistical methods. Therefore, it is crucial to develop and refine stakeholder engagement strategies in the context of CER that employs real-world data and sophisticated computational techniques.

Our approach showcases the feasibility and benefits of involving diverse stakeholder groups in real-world data studies. Similarly, a recent paper on stakeholder engagement in a comparable research context to ours found that the participation of clinician and patient stakeholders was both feasible and enhanced the quality of the research [29]. However, this study did not include other important stakeholder perspectives (e.g., representatives from payer organizations or professional societies), who likely hold different knowledge, views, and priorities than patients and clinicians (and each other). Notably, the inclusion of leaders from professional advocacy groups and payers in our study represents a shift from typical practice. A 2014 systematic review of stakeholder engagement models indicated that patients were by far the most commonly engaged group among included studies, followed by modest engagement of clinicians, and infrequent engagement of other stakeholders across the healthcare system [10]. A more recent review, limited to PCORI-funded studies, found significant patient and clinician involvement, with each group included as partners in nearly 90% of included studies. However, engagement with other stakeholders like payers, health systems, professional societies, and subject matter experts remained low [29].

By including a broader range of stakeholders, we sought to understand and integrate their diverse perspectives and priorities in our research. This integration is crucial because the choice of T2D pharmacotherapy is driven not only by patient and clinician preferences but also by professional society recommendations and insurance formularies. Our goal was to align these diverse perspectives to ensure that professional societies can recommend, clinicians can prescribe, payers can reimburse, and patients can receive – with minimal administrative and financial burdens – the most effective and safe T2D medications.

In addition, stakeholders have historically been more heavily engaged in earlier stages of research (i.e., evidence prioritization) than in later activities such as interpretation and application of findings [10]. While our PSAG members were substantively involved in early stage research activities such as developing the study (e.g., framing the research topic, developing specific aims, specifying methods) and selecting outcomes to be studied, an inclusive, stakeholder-engaged approach also ensures that diverse perspectives are considered throughout. In our experience, stakeholder involvement was impactful across the full spectrum of the research process as performed to date, influencing meaningful decisions related to study implementation and analysis. Moreover, PSAG member survey results indicated a strong desire among members to be involved in future activities, especially in interpreting research findings and communicating and disseminating results to various groups. As such, PSAG members – as appropriate based on their interest and availability – have been included in all dissemination activities to date, including conference presentations and peer-reviewed publications.

Finally, engaging patients and other stakeholders in studies like ours that center on analyses of real-world data may pose specific challenges due to the complex statistical modeling and technical concepts typically involved. Although our PSAG member survey indicated high levels of satisfaction with how the study’s objectives were communicated, the technical nature of its methodology did present some challenges to PSAG engagement. Notably, this issue wasn’t confined to patient members, as other stakeholders without extensive research training struggled to grasp some details of the research design and processes. This gap in understanding risked limiting their ability to provide comprehensive feedback and engage effectively with the study. To overcome this potential issue, the study team deliberately and repeatedly attempted to describe the research methods in a manner that was accessible and straightforward, demonstrating that bridging this communication gap and finding a common language is possible within the relatively short lifespan of a grant.

Several limitations must be considered. First, the selected nature and small number of PSAG members limit the representativeness of the group and the generalizability of the findings to other studies. Next, the engagement process itself – specifically, the limited frequency of formal meetings – may have restricted the depth and breadth of stakeholder input. However, PSAG members repeatedly exchanged feedback and ideas with the research team between meetings over email or in small group, ad hoc meetings. Finally, findings from the assessment survey are based on PSAG member perceptions at an early stage of the grant period and may have failed to capture differences in member satisfaction and goals that emerged at a later point. However, we plan to field a follow-up survey closer to the grant’s conclusion. Another limitation of the survey was that it was not conducted anonymously, which may have influenced the objectivity of responses. The decision to make the survey non-anonymous was intentional, as it allowed the study team to address specific feedback directly and tailor responses to individual needs. However, we recognize that this approach may have affected members’ willingness to provide candid feedback.

## Conclusions

To date, best practices for engaging stakeholders in comparative effectiveness studies using real-world or administrative data have not been articulated. Our study suggests that an inclusive, stakeholder-engaged approach not only enriches the research process but also ensures that diverse perspectives are integrated into both the study design and its implementation. By consistently refining communication strategies and broadening the ways in which stakeholders can be involved, researchers can more effectively tackle the complex challenges associated with T2D medication management and significantly improve the impact and relevance of findings.
